# Supplementary material for: Multi-omics analysis identifies therapeutic vulnerabilities in triple-negative breast cancer subtypes
Source: Nat Commun. 2021 Nov 1;12:6276. doi: 10.1038/s41467-021-26502-6 (PMC8560912; doi:10.1038/s41467-021-26502-6)
Supplement: Supplementary file 1 — Supplementary Information [file 41467_2021_26502_MOESM1_ESM.pdf]

Multi-omics analysis identifies therapeutic vulnerabilities in triple-negative breast cancer subtypes

Supplementary Information

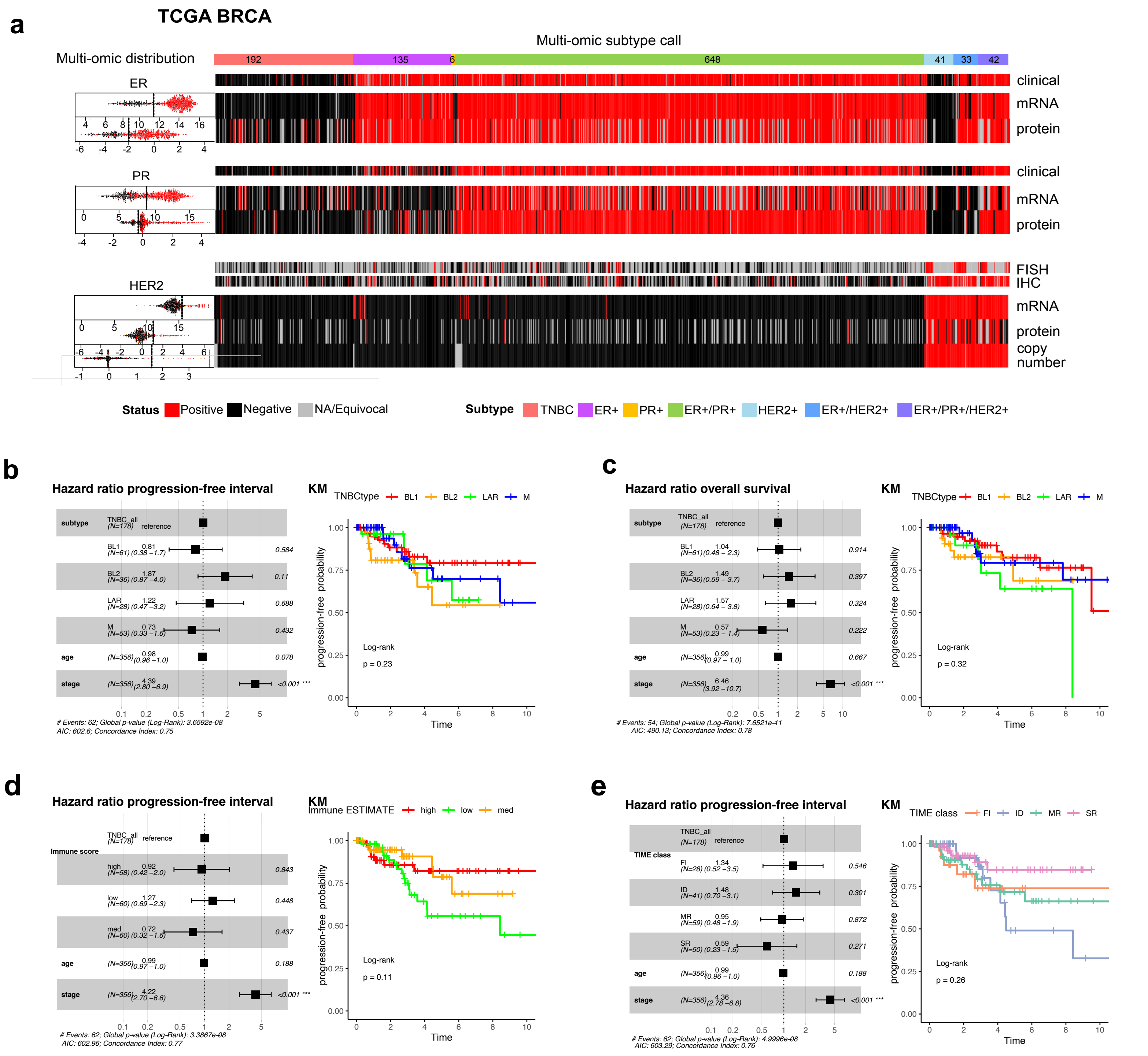

Supplementary Fig. 1. Pathology-guided multi-omic identification of TNBC samples. a, Sina plots show the distribution of ER, PR or HER2 mRNA and protein expression or *ERBB2* copy number for individual breast cancer (BRCA) patient tumors in TCGA. Data points are colored clinical assay results (positive, red; negative, black; NA and equivocal, grey). Dotted lines indicate cutoffs for inferred genomic calls. Colorbars show individual tumors colored by clinical results or multi-omic distribution calls (positive, red; negative, black) stratified into clinical subtypes. Hazard-ratio plots and Kaplan-Meier plots for **b,** progression-free survival and **c,** overall survival in TNBC subtypes relative to all TNBC adjusted for age and tumor stage. Hazard-ratio plots and Kaplan-Meier plots for TNBC tumors stratified by **d,** immune Xcell immune score tertile or **e,** tumor microenvironment (TIME). FI, fully inflamed; ID, immune desert; MR margin-restricted; and SR, stroma-restricted. Error bars (b-e) represent 95% confidence interval.m Global log-rank p-values indicated on charts.

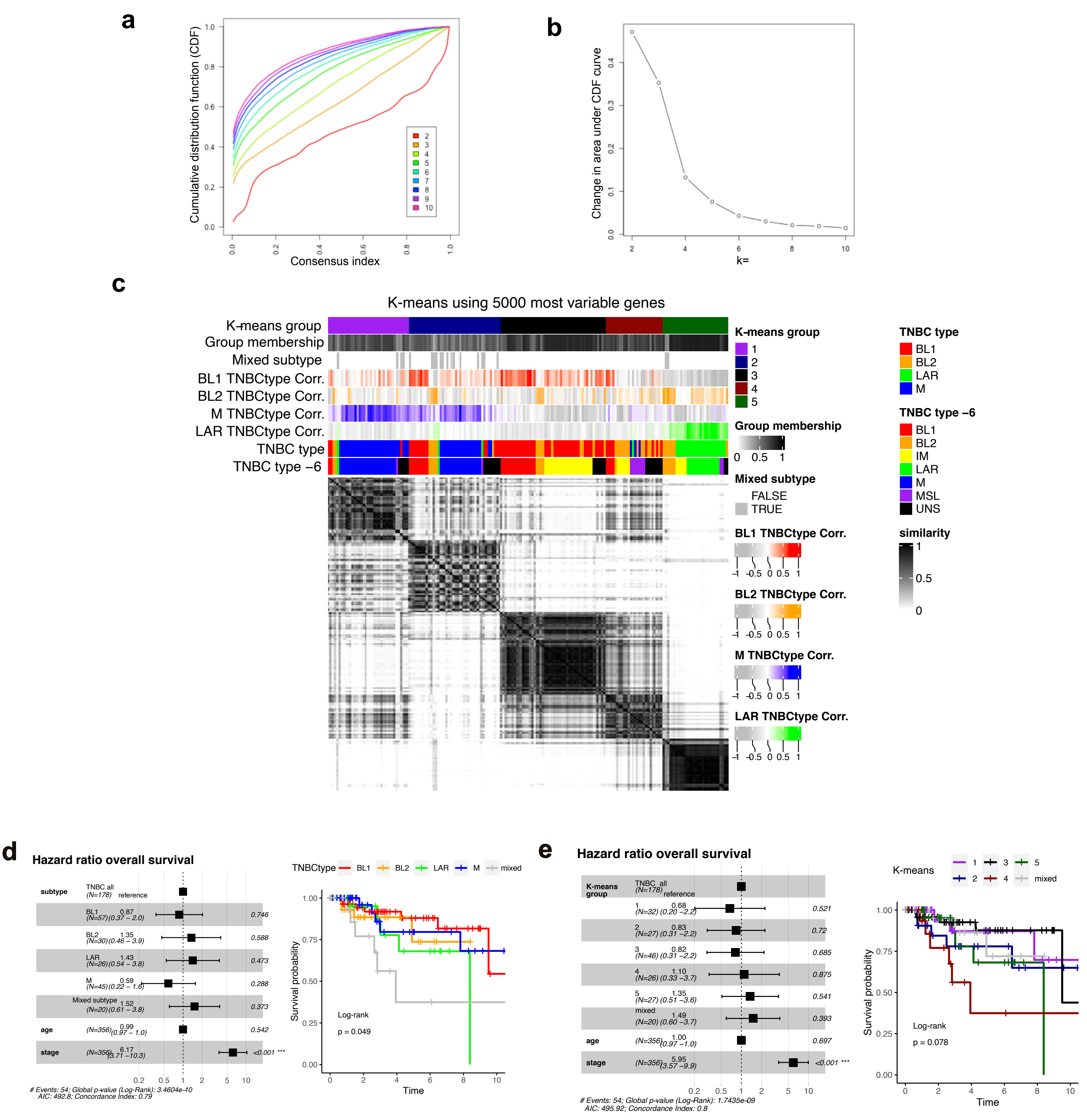

Supplementary Fig. 2. Unbiased k-means consensus clustering identifies five subtypes. Unbiased k-means consensus clustering was performed on the 192 TNBC samples in TCGA. **a,** The empirical cumulative distribution function (CDF) plot shows the functions of the consensus matrix for each k[1-10] **b,** Relative change in area under the CDF curve comparing k and k−1. The number of clusters is decided when any further increase in cluster number (*k*) does not lead to a corresponding marked increase in the CDF area **c,** Heatmap of TNBC samples similarity. Annotations show: 1) k-means group, 2) membership correlation, 3) mixed subtype assignment, 4) the continuous correlation with TNBCtype subtypes (BL1, BL2, M and LAR), 5) TNBC final assignment considering 4-class TNBC subtypes (BL1, BL2, M and LAR), and 6-class TNBC subtypes (BL1, BL2, M, LAR, MSL and IM) 6) TNBC Consensus clustering (CC) groups results from the unsupervised algorithm k-means (k=5) of the 5000 most variable genes. Forrest plots shows hazard ratio for overall survival event compared to all TNBC and Kaplan-Meier plot for overall survival stratified by **d**, TNBC subtype or **e**, k-means consensus cluster. Error bars (d and e) represent 95% confidence interval. Global log-rank p-values indicated on charts.

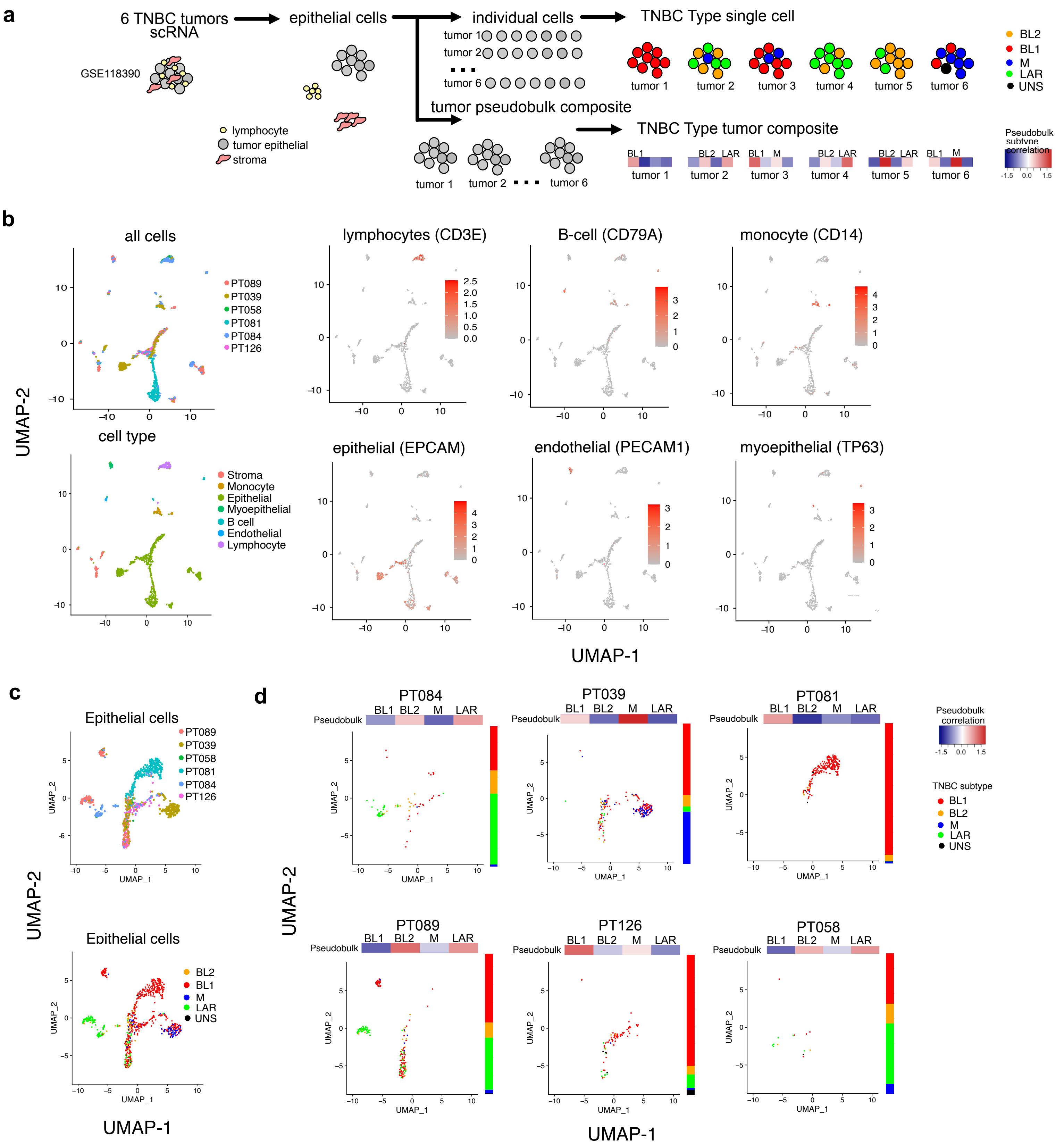

Supplementary Fig. 3. Analysis of single-cell RNA-seq (scRNA) reveals intra-tumor TNBC subtype heterogeneity. a, Schema for evaluating TNBC subtype composition from individual cells and pseuobulk composite for each tumor from scRNA sequencing **b**, UMAP plots from six individual TNBC tumors (GSE118390) colored by sample or expression of specific cell lineage markers representing lymphocyte (CD3E), B-cells (CD79A), monocyte (CD14), epithelial (EPCAM), endothelial (PECAM1) and myoepithelial (TP63) cell populations. **c**, UMAP plots for all epithelial cells identified in b, renormalized and colored by sample or TNBC subtype. **d**, Individual U-map plots for epithelial cells from each patient with individual cells colored by TNBC subtype. Side colorbars represent quantification of individual cells by subtype. Colorbars above plots indicate subtype correlation strength for each subtype from the pseudobulk composite of the integrated expression of all cells for each patient tumor.

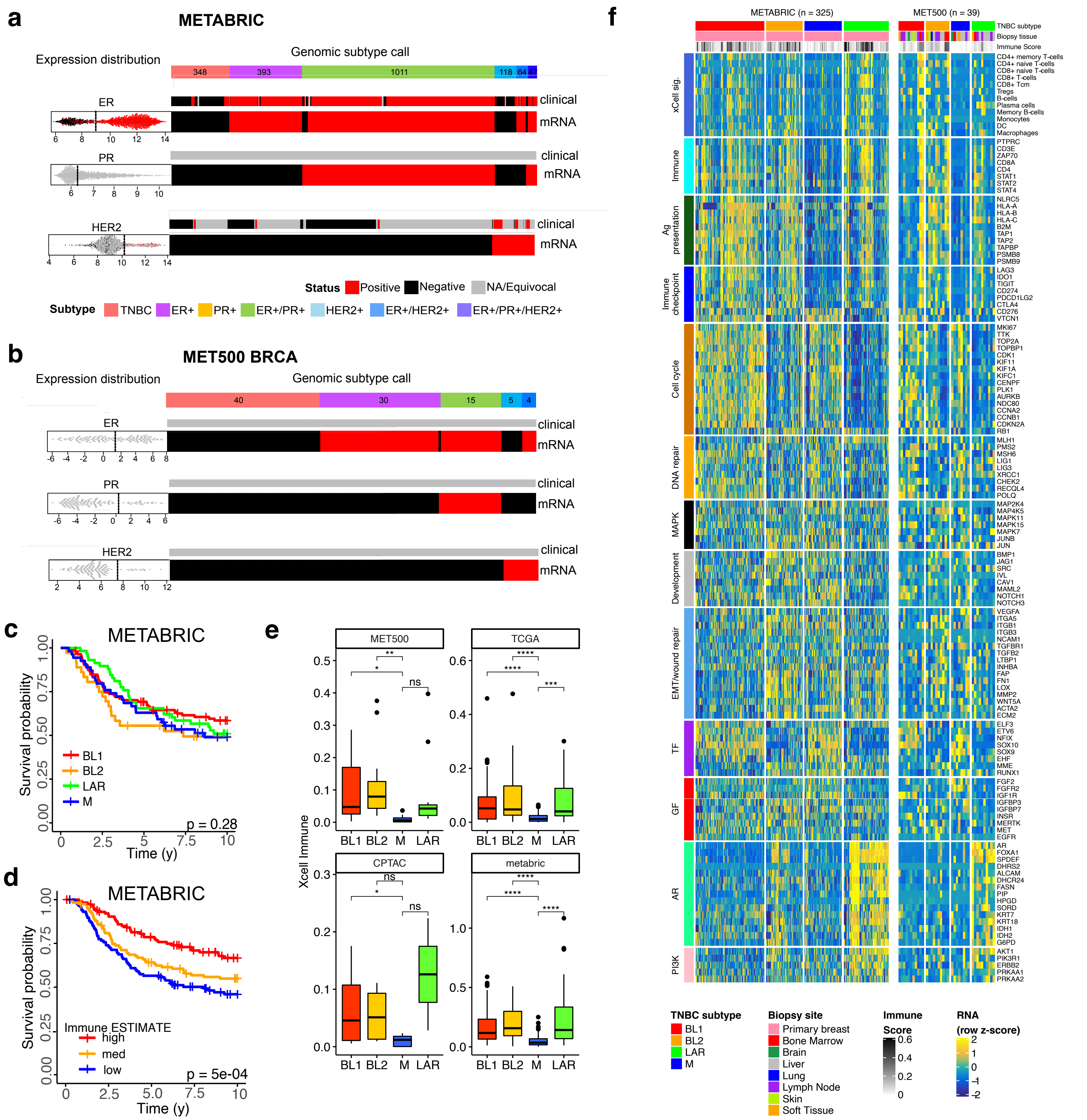

Supplementary Fig. 4. Identification and characterization TNBC tumors in METABRIC and MET500. Sina plots show the distribution of ER, PR or HER2 mRNA expression for individual breast cancer (BRCA) patient tumors in the **a,** METABRIC or **b,** MET500 dataset. Data points are colored clinical assay results (positive, red; negative, black; NA and equivocal, grey). Dotted lines indicate cutoffs for inferred genomic calls. Colorbars show individual tumors colored by clinical results or expression distribution calls (positive, red; negative, black) stratified into clinical subtypes. Kaplan-Meier plots for overall survival of METABRIC TNBC patients stratified by **c,** TNBC subtype or **d**, immune score (ESTIMATE) tertials. Indicated p-values from log-rank test. **e,** Boxplots show distribution of inferred immune score (xCell) by TNBC subtype in METABRIC (n=348), TCGA (n=183), CPTAC (n=27) and MET500 (n=39) cohorts. Boxplot elements: center line, median; box limits, upper and lower quartiles; whiskers, 1.5× interquartile range. P-value was determined using a two-sided t test, *P < 0.05, **P < 0.005, ***P < 0.0005, ****P < 0.00005. See source file for raw p-values. **f,** Heatmap shows gene expression (LFC >1, FDR p-value <0.05) from modified T-test from limma package by pathways and inferred immune composition (significantly enriched cell types identified by xCell) from primary TNBC (METABRIC) and metastatic TNBC (MET500) cohorts stratified by TNBC subtype.

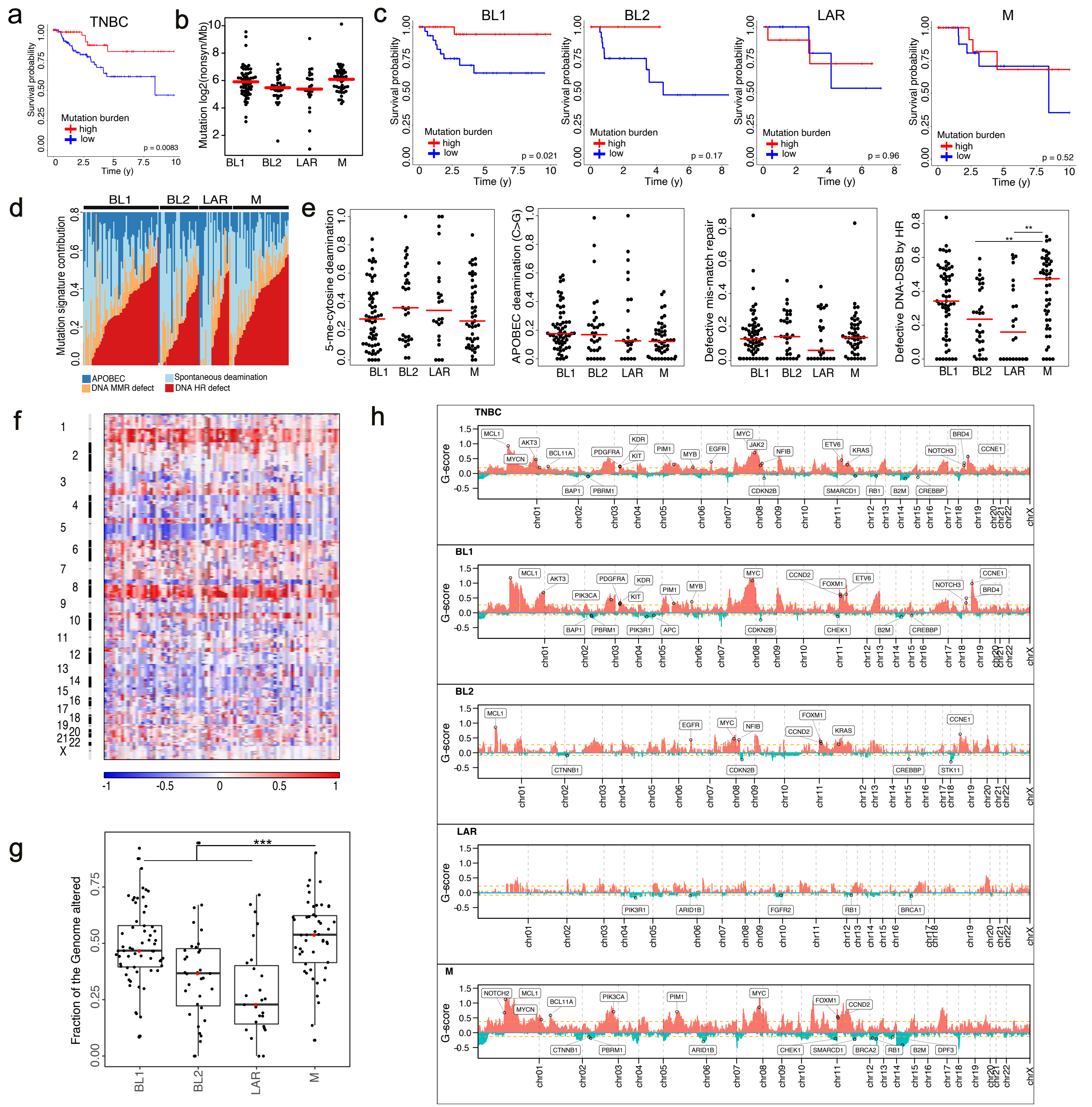

Supplementary Fig. 5. TNBC subtypes display unique mutational and copy number patterns. a, Kaplan-Meier survival plot show progression-free interval (PFI) for all TCGA TNBC patients stratified by tumor mutational burden (TMB) high (>1.5mut/Mb) or low (<1.5mut/Mb). Log-rank test. **b,** Sina plot shows mutation load for individual TNBC patients from TCGA stratified by subtype. Two-tailed student t-test was used for statistical analyses. **c,** Kaplan-Meier survival plots show PFI for TNBC subtypes stratified by tumor mutational burden. Indicated p-valued from Log-rank tests. **d,** Plot shows distribution of mutational signatures for individual TCGA tumors stratified by TNBC subtype. **e,** Sina plots show distribution and median (red line) for each mutational signature by TNBC subtype. Significance determined by Tukey's honest significant difference test. Raw p-values in source file. **f,** Heatmap shows significant copy number variation for all TCGA TNBC determined by GISTIC 2.0 permutation test. **g,** Boxplot shows fraction of the genome altered (FGA) calculated from segment level copy number data from TCGA (n=167) stratified by subtype. Boxplot elements: center line, median; box limits, upper and lower quartiles; whiskers, 1.5× interquartile range.Significance determined by unpaired two-tailed T-test (p=0.001399). **h,** Plots show G-score (GISTIC2) for recurring amplifications and deletions for all TNBC tumors and those enriched in each TNBC subtype. Peaks and valleys annotated with known oncogenes and tumor suppressor genes, respectively, significantly (FDR < 0.25, orange dotted line) enriched within each subtype. *, p<0.05; **, p<0.01; ***, p<0.001.

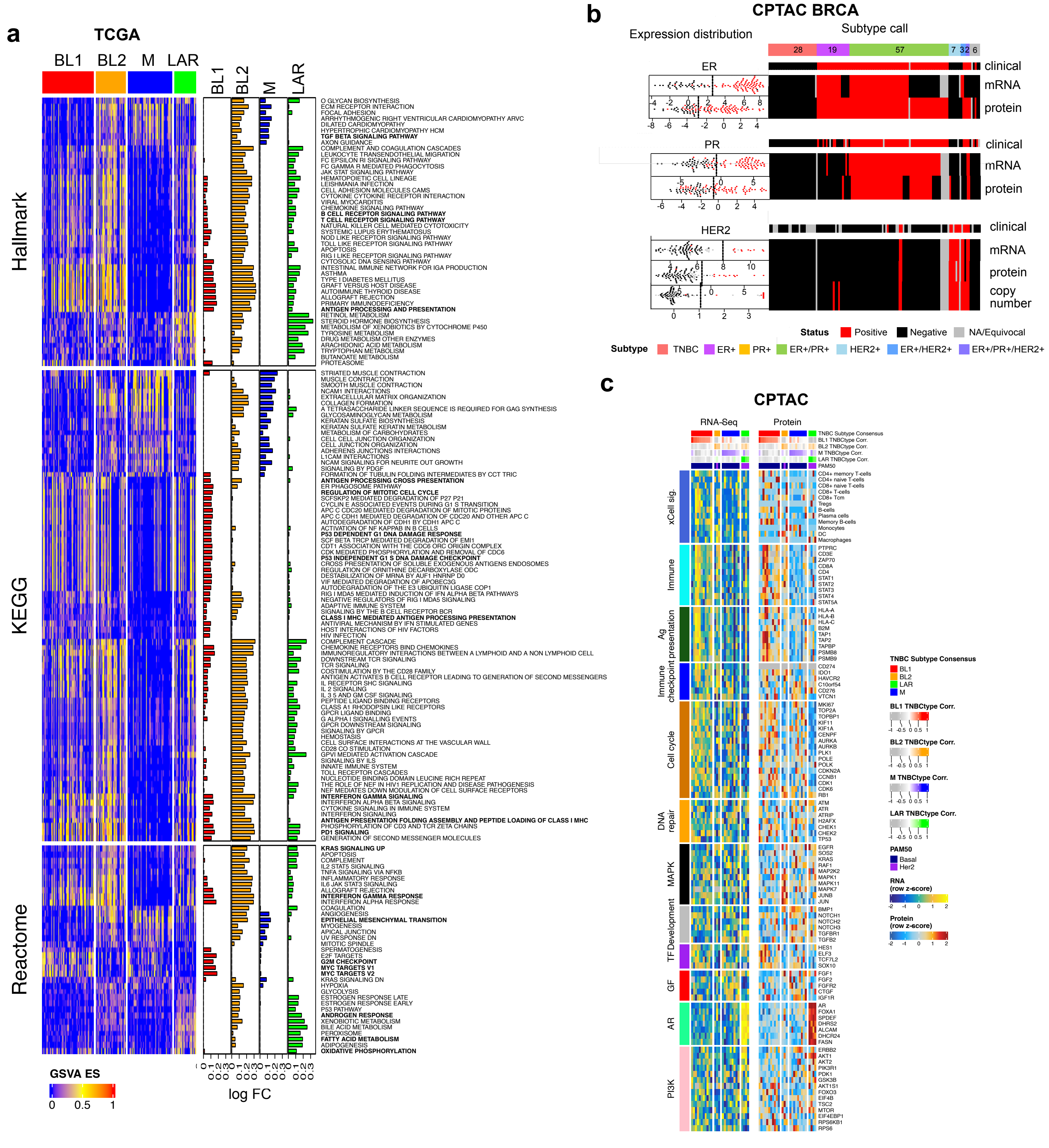

Supplementary Fig. 6. RNA and protein analysis identify key pathways in TNBC subtypes a, Heatmap shows gene set variation analysis (GSVA) enrichment scores (ES) in Hallmark, KEGG and Reactome pathways significantly (FDR <0.03) enriched in individual TCGA TNBC tumors. Adjacent barplots show aggregated log fold change for all tumors of a given subtype. Pathways of interest are indicated in bold. **b,** Sina plots show the distribution of ER, PR or HER2 mRNA and protein expression or *ERBB2* copy number for individual breast cancer (BRCA) patient tumors in CPTAC. Data points are colored clinical assay results (positive, red; negative, black; NA and equivocal, grey). Dotted lines indicate cutoffs for inferred genomic calls. Colorbars show individual tumors colored by clinical results or expression distribution calls (positive, red; negative, black) stratified into clinical subtypes. **c,** Relative protein and RNA expression for TNBC patients in CPTAC stratified by subtype and pathways.

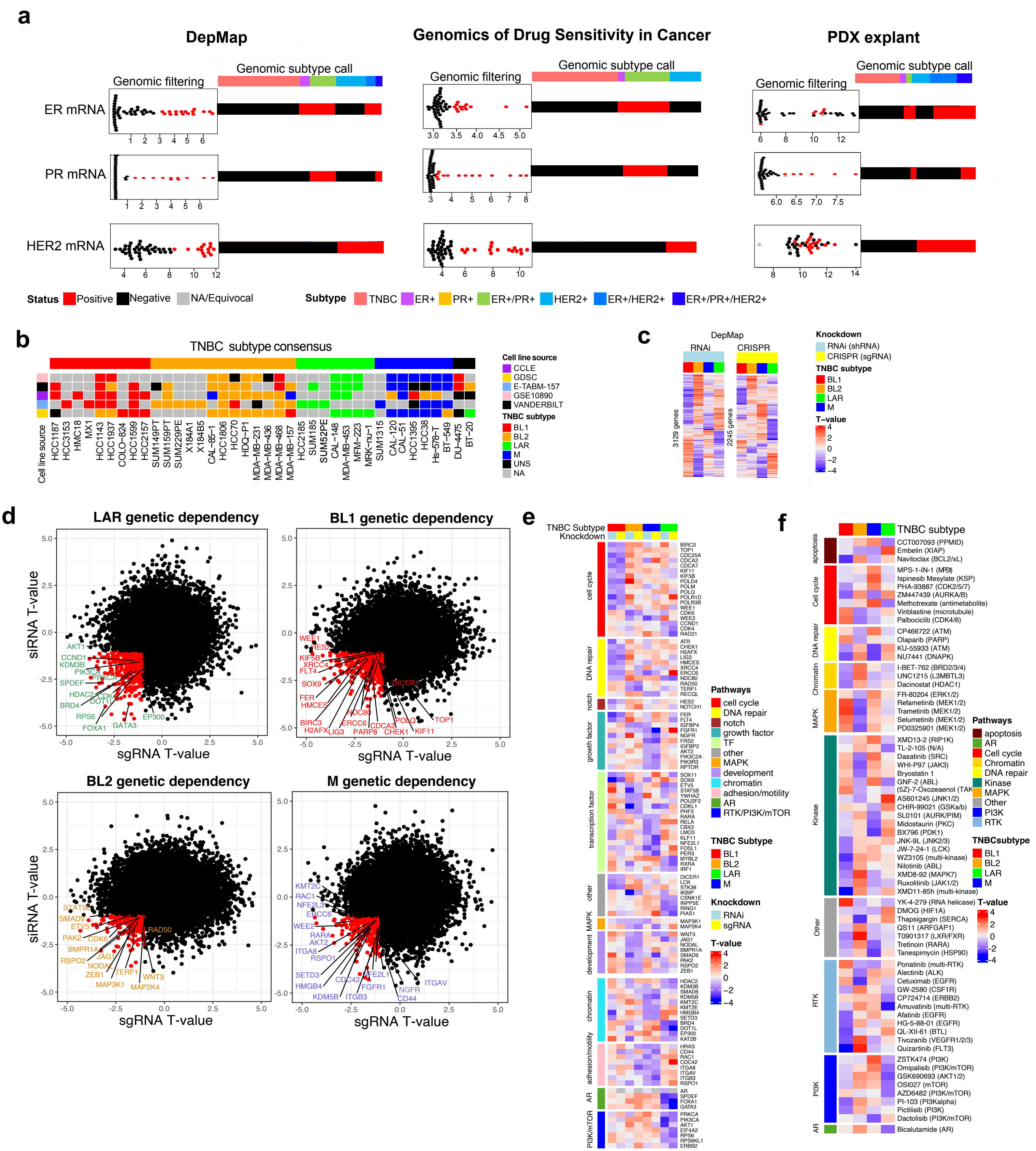

Supplementary Fig. 7. TNBC cell line characterization and genetic/pharmacological dependencies. a, Sina plots show mRNA distribution for ER, PR and HER2 across breast cancer cell lines in DepMap, Genomics of Drugs Sensitivity in Cancer and PDX explant cohorts. Barplots show genomic-guided stratification of cell lines. Colorbars show individual models colored by genomic distribution calls (positive, red; negative, black; NA/equivocal, grey). **b,** Barplot shows consensus TNBC subtyping from five datasets (CCLE, GDSC, E-TABM-157, GSE10890 and Vanderbilt). **c,** Heatmap shows significant genetic dependencies by TNBC subtype for genes depleted in TNBC cell lines from whole-genome RNAi and CRISPR (sgRNA) screens (DepMap). **d,** Starburst plot shows consistent genetic dependencies (T-values) between siRNA and CRISPR screens with genes-of-interest indicated in each subtype plot. Heatmap of select **e,** genetic and **f,** pharmacologic dependencies of TNBC cell lines by subtype.

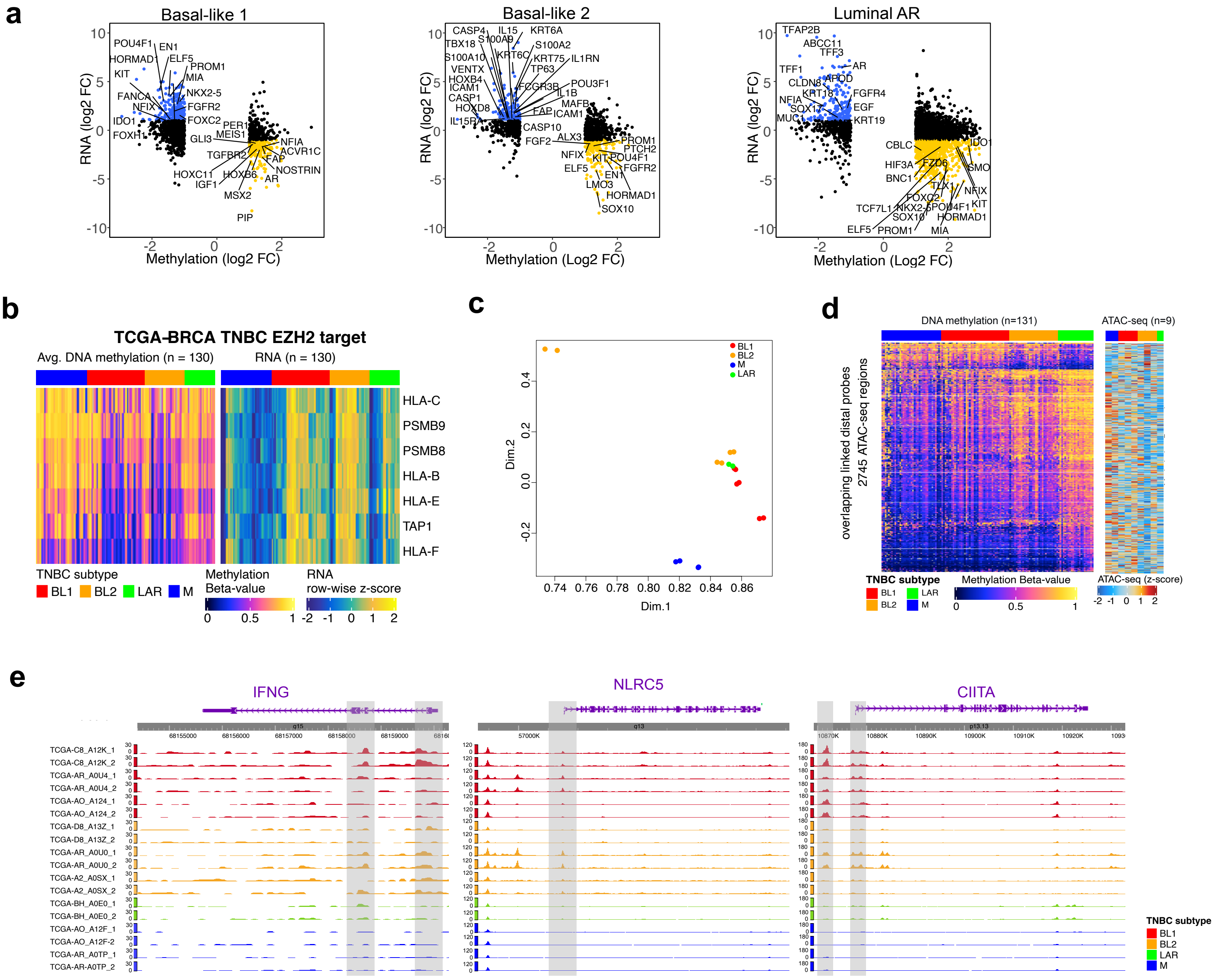

Supplementary Fig. 8. DNA methylation and chromatin accessibility differ by subtype. a, Starburst plots show gene expression and DNA methylation <3kb from promoter regions of corresponding genes. Significantly hypo- and hyper-methylated promoter regions are colored in blue and yellow respectively, with genes of interest labeled in the plot. **b,** Heatmaps show average DNA methylation (β-value) and corresponding RNA expression for the indicated antigen presentation genes. **c,** Principal component analysis plot shows variance for ATAC-seq for TCGA TNBC tumors colored by subtype. **d,** Heatmaps show chromatin accessibility (ATAC-seq) at distal CpGs methylation sites (β-value) for putative target gene-methylation in TCGA by TNBC subtype. **e,** Individual chromatin accessibility (ATAC-seq) tracks for regions in *IFNG*, *NLRC5* and *CIITA*. Highlighted boxes indicate promoter region peaks absent in mesenchymal tumors.

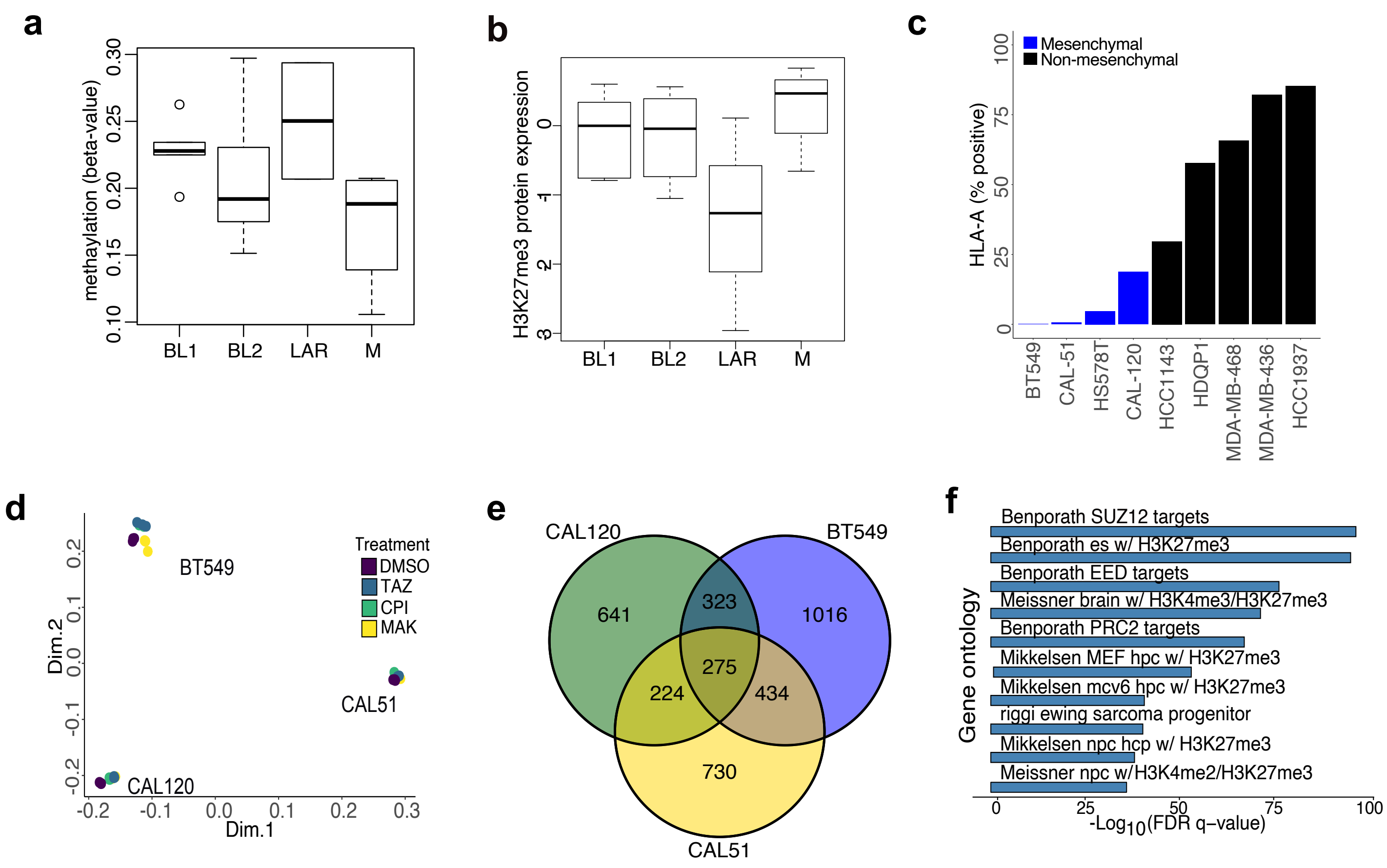

Supplementary Fig. 9. Mesenchymal TNBC cell lines displayed decreased global methylation, increased H3K27me3 and EZH2 inhibition increases PRC2 target expression. Boxplots show **a,** Global DNA methylation (average β-value, n=22) and **b,** H3K27me3 across TNBC cell lines (n=20) in the CCLE stratified by subtype. Boxplot elements: center line, median; box limits, upper and lower quartiles; whiskers, 1.5× interquartile range. **c,** Barplot shows IHC quantification of MHC-I positive (% positive) cells across TNBC cell lines in a tissue microarray. **d,** PCA plot shows gene expression variability of TNBC cell lines 5 days after treatment with PRC2 inhibitors. TAZ=tazemetostat, CPI=CPI-1205 and MAK=MAK-683. **e,** Venn diagram shows differentially expressed transcripts that increased with EZH2 inhibition in BT549, CAL51 and CAL120 cells. **f,** Barplot shows most significantly (-log10 p-value) enriched pathways (MsigDB C2:CGCP) for genes increased in expression from union of differentially expressed transcript from all cell lines in e.

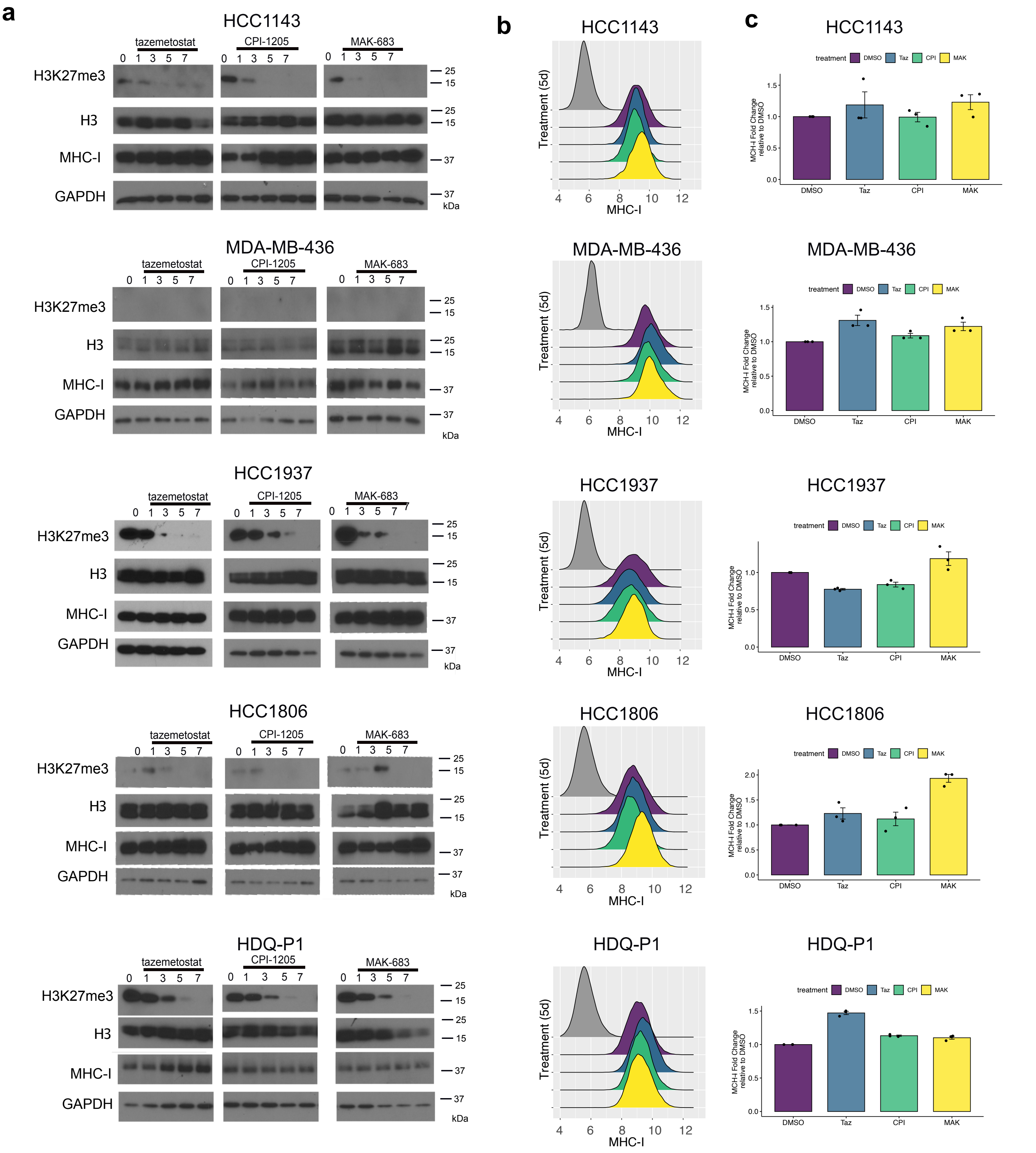

Supplementary Fig. 10. PRC2 inhibition decreases H3K27me3 without increasing MHC-I expression in non-mesenchymal TNBC cells. a, Immunoblots show H3K27me3 and MHC-I protein expression in HCC1143, MDAMB436, HCC1937, MDAMB468, and HDQP1 at 1, 3, 5, 7 days after a single 10 µM treatment with either tazemetostat (TAZ), CPI-1205 (CPI) or MAK-683 (MAK). **b,** Histograms show distribution and **c,** quantification mean fluorescence of cell-surface MHC-I protein expression 5 days after a 10 µM treatment with the indicated PRC2 inhibitors. Error bars were determined from three independent experiments.

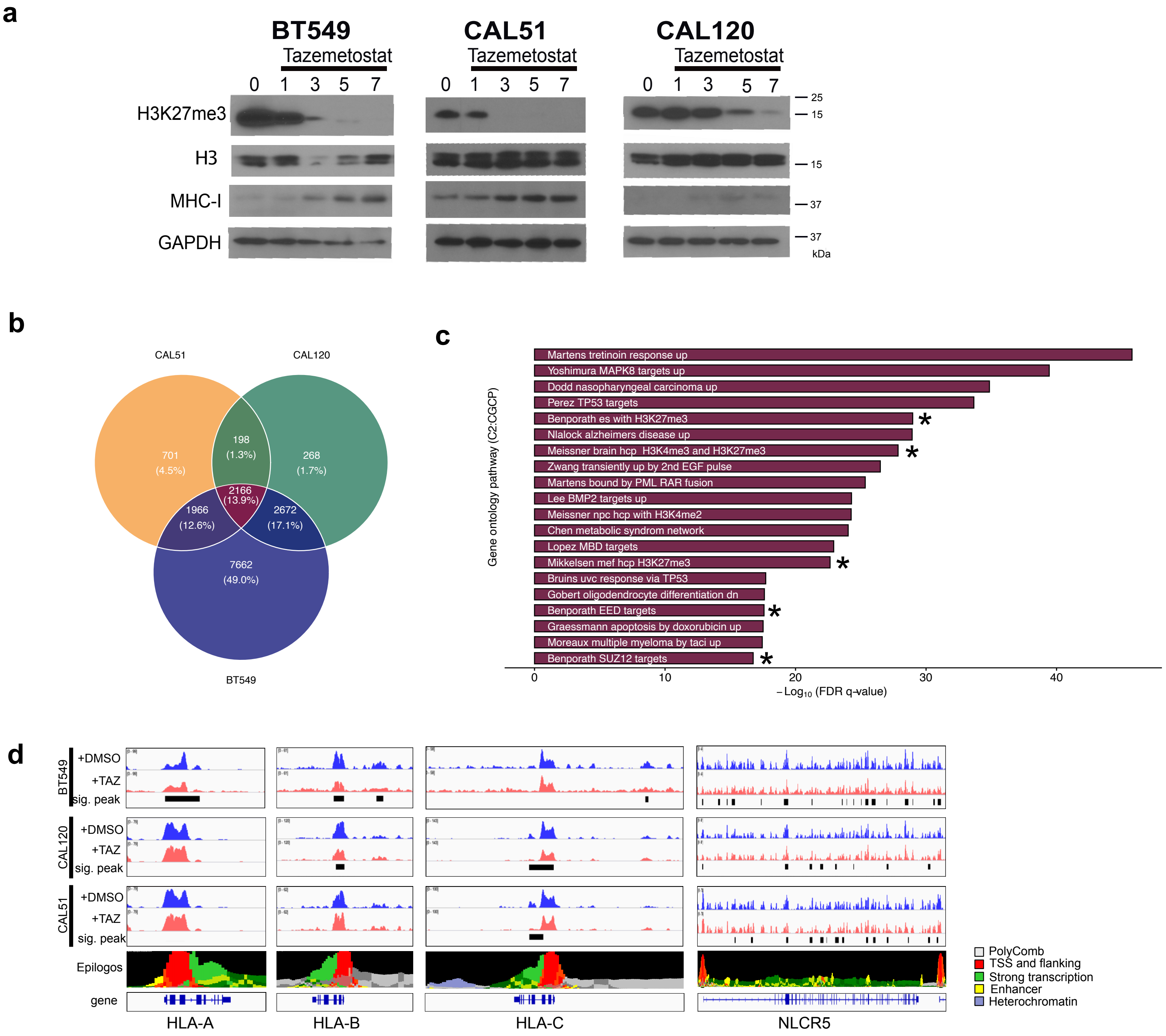

Supplementary Fig. 11. EZH2 inhibition restores MHCI expression and decreases H3K27me3 promoter occupancy at EZH2 targets and MHCI locus. **a,** Immunoblots show H3K27me3 and MHC-I protein expression in mesenchymal TNBC cells at 1, 3, 5, 7 days treated with 1 μM tazemetostat. Immunoblots are representative of two independent experimetns. **b,** Venn diagram shows overlap of significantly decreased H3K27me3 ChIP-seq peaks in promoter regions from BT549, CAL120 and CAL51 cells treated with 1 μM tazemetostat for four days. **c,** Significantly enriched gene sets (C2:CGCP) form the union of H3K27me3 peaks in b. * indicate pathways associated with PRC2. **d,** Representative genomic tracks for H3K27me3 at indicated loci and significantly decreased peaks (black bars below tracks) for ChIP-seq performed in the indicated cell lines treated with DMSO (blue) or tazemetostat (red). Epilogos tracks from corresponding loci showing summation of chromatin states from hundreds of epigenomes.

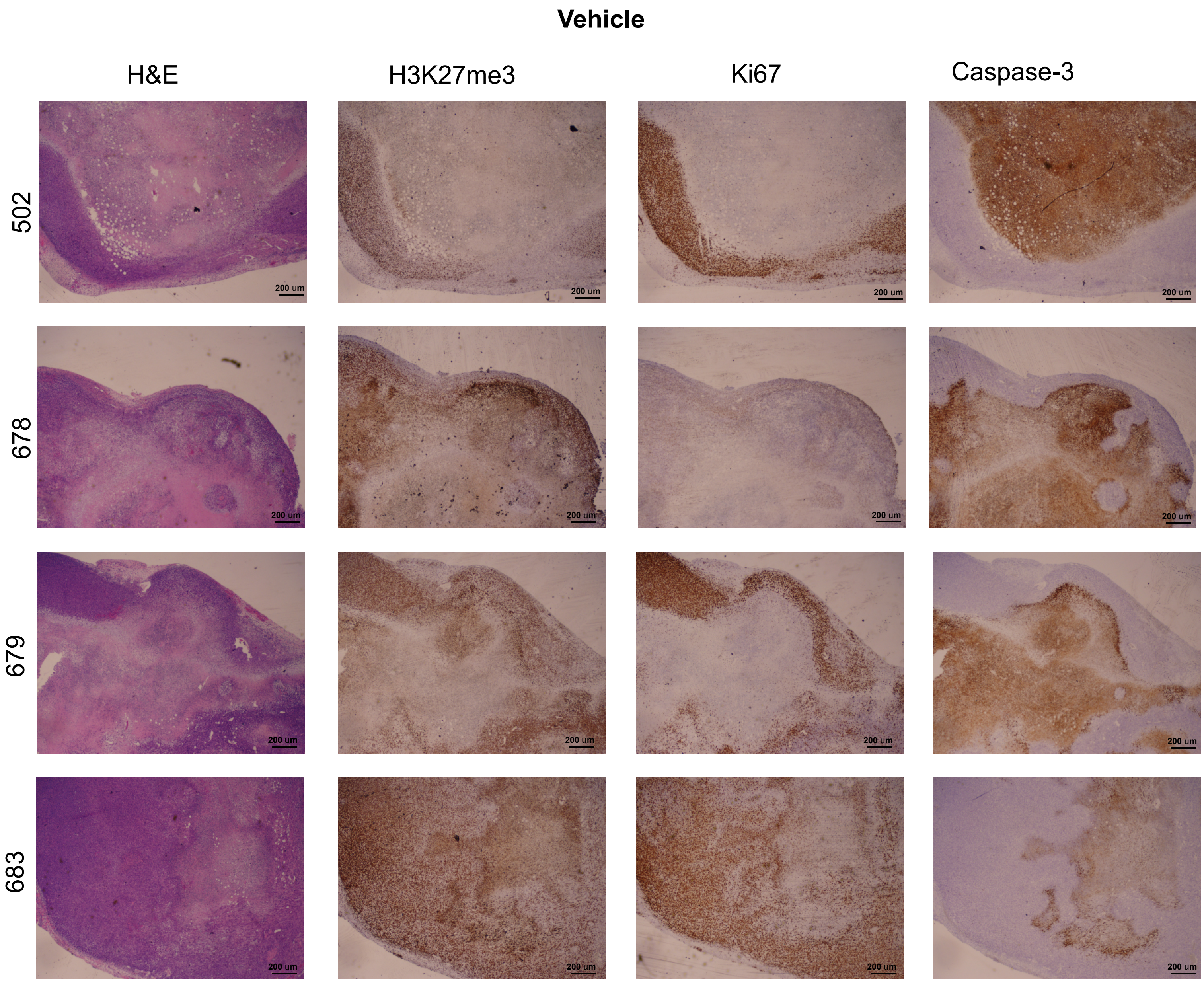

Supplementary Fig. 12a. Immunohistochemistry of H3K27me3, Ki67 and caspase-3 in xenograft tumors from mice treated with vehicle. Images from serial sections of individual 4T1 tumors (rows) stained for H&E or H3K27me3, Ki-67 and cleaved caspase-3. Images are representative of tumors from four independent experiments.

Paclitaxel

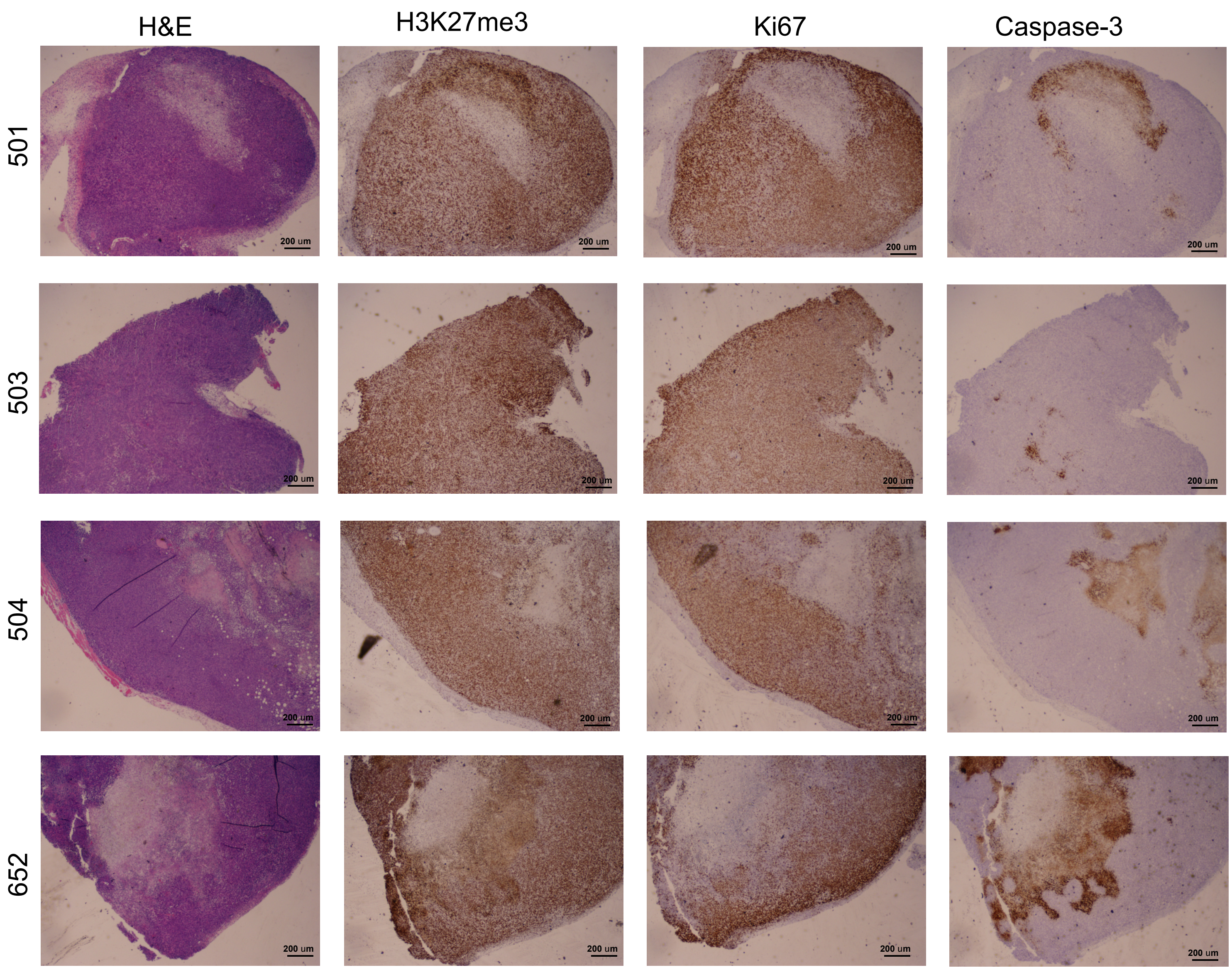

Supplementary Fig. 12b. Immunohistochemistry of H3K27me3, Ki67 and caspase-3 in xenograft tumors from mice treated with paclitaxel. Images from serial sections of individual 4T1 tumors (rows) stained for H&E or H3K27me3, Ki-67 and cleaved caspase-3 .Images are representative of tumors from four independent experiments.

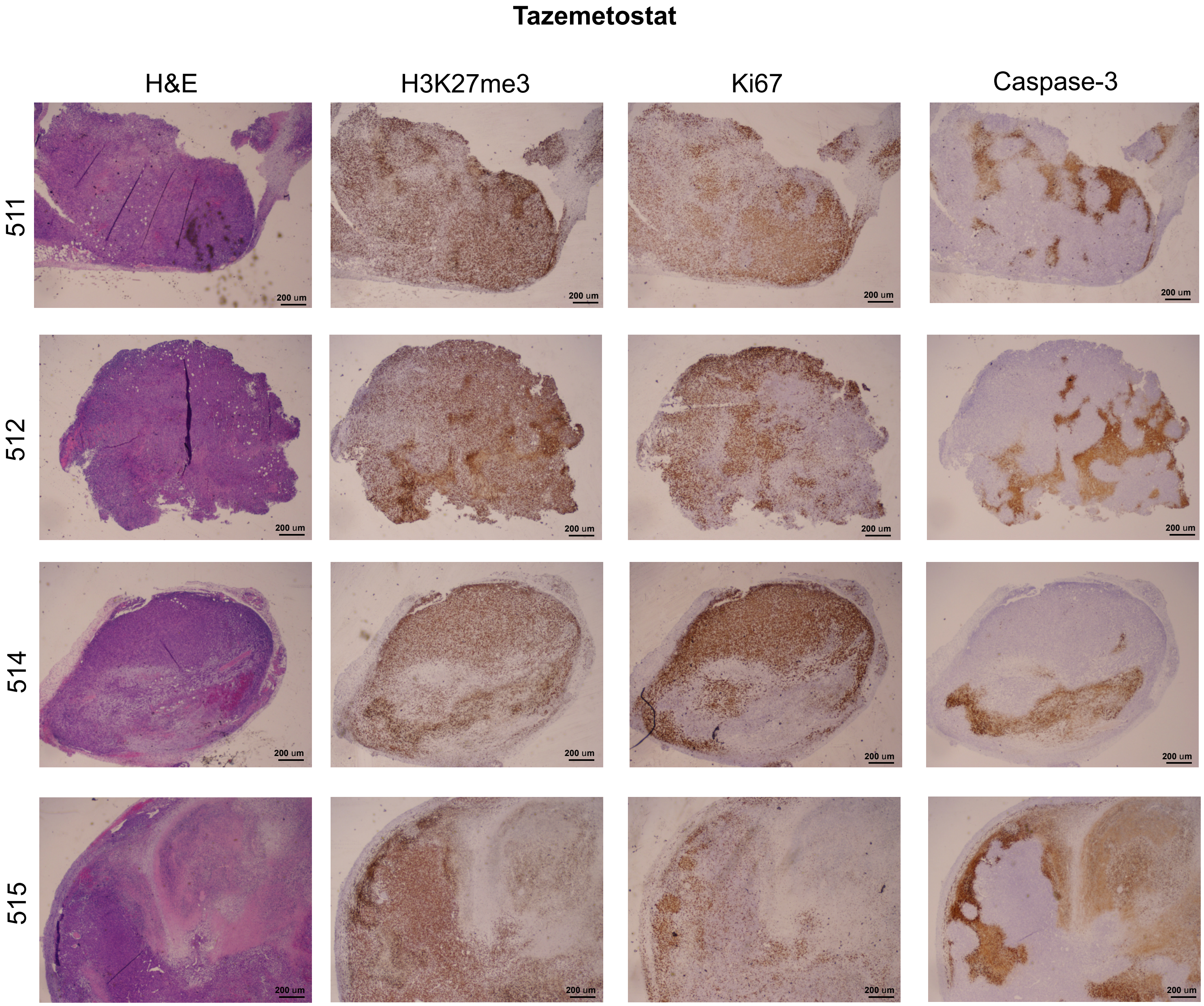

Supplementary Fig. S12c. Immunohistochemistry of H3K27me3, Ki67 and caspase-3 in xenograft tumors from mice treated with tazemetostat. Images from serial sections of individual 4T1 tumors (rows) stained for H&E or H3K27me3, Ki-67 and cleaved caspase-3. Images are representative of tumors from four independent experiments.

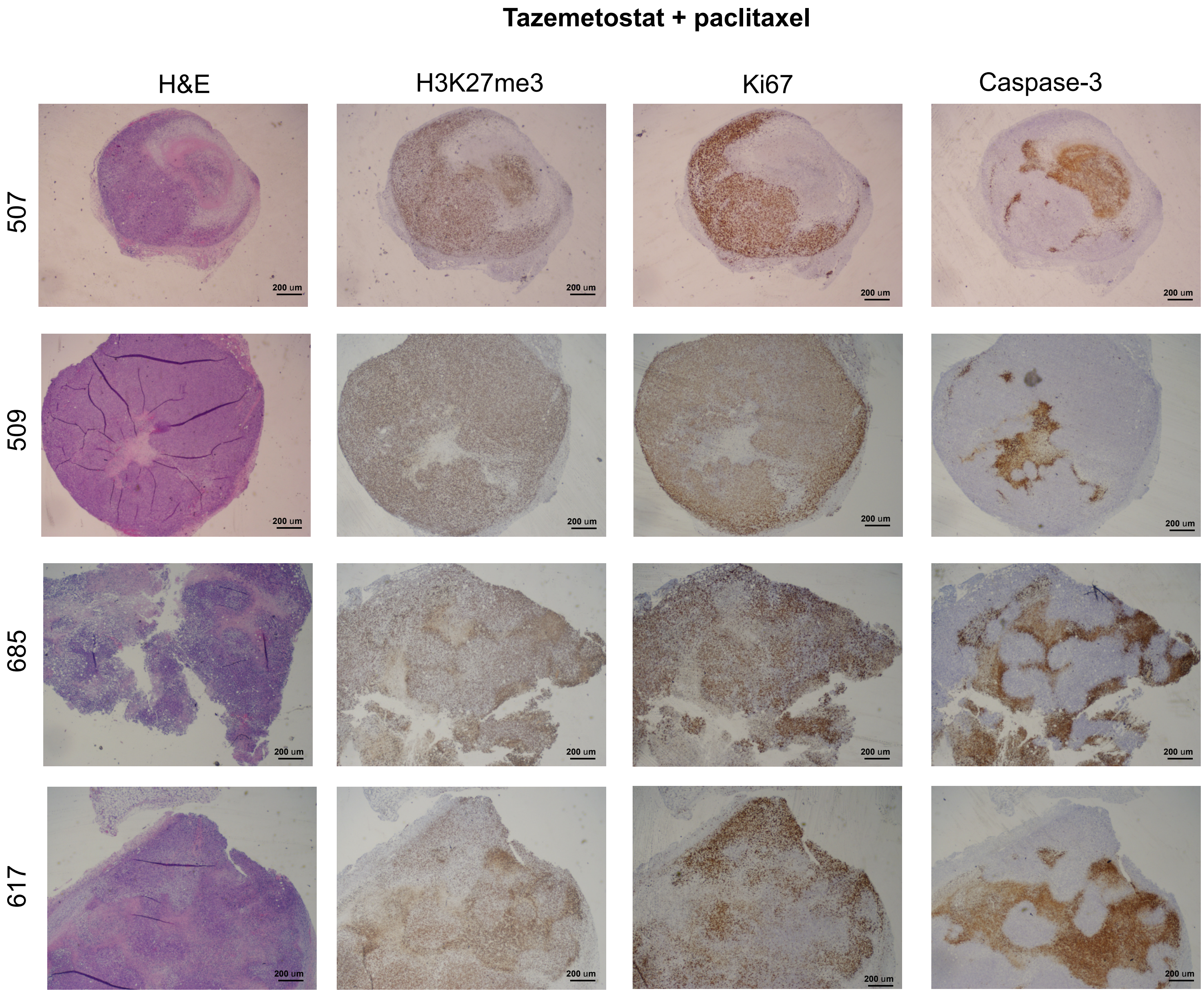

Supplementary Fig. 12d. Immunohistochemistry of H3K27me3, Ki67 and caspase-3 in xenograft tumors from mice treated with tazemetostat + paclitaxel. Images from serial sections of individual 4T1 tumors (rows) stained for H&E or H3K27me3, Ki-67 and cleaved caspase-3. Images are representative of tumors from four independent experiments.

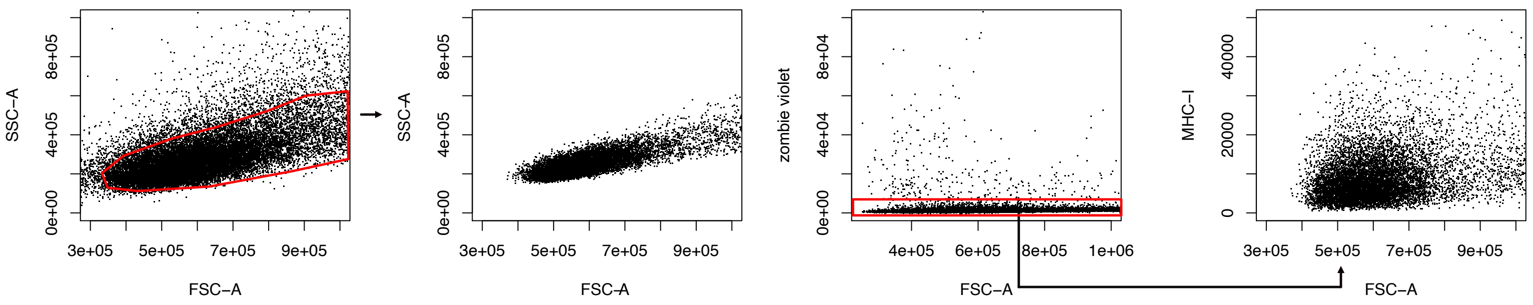

Supplementary Fig. 13. **Gating strategy to determine the mean fluorescence intensity of MHC-I expression.** FSC/SCC exclusion of debris. Negative staining for Zombie Violet to remove dead cells, positive staining for PE-conjugated anti H2_Kd (mouse) or HLA-A/B/C (human). Gating strategy relevant for figures 5c, 5j, 7b and S10b.
