# Supplementary material for: Multi-omics analysis identifies therapeutic vulnerabilities in triple-negative breast cancer subtypes
Source: Nat Commun. 2021 Nov 1;12:6276. doi: 10.1038/s41467-021-26502-6 (PMC8560912; doi:10.1038/s41467-021-26502-6)
Supplement: Supplementary file 3 — Description of Supplementary Data [file 41467_2021_26502_MOESM3_ESM.pdf]

Description of Additional Supplementary Files:

Supplementary Data 1 (Microsoft Excel format): ER, PR and HER2 inference by genomic data and clinical results. a, TCGA: TCGA barcode identifiers, pathology and genomic calls for ER, PR and HER2. **b,** CPTAC: CPTAC barcode identifies, pathology and genomic calls for ER, PR and HER2. **c,** METABRIC: METABRIC IDs, pathology calls, ER, PR and HER2 expressions and genomic calls. **d,** MET500:MET500 identifiers, biopsy site, ER, PR and HER2 expression and genomic calls.

Supplementary Data 2 (Microsoft Excel format): TNBC subtype clinical information and signatures.
a, TCGA: TCGA barcode identifiers, clinical, TNBC subtype correlation, mutation signature, mutation complexity (MATH), immune classification, immune ESTIMATE, Xcell, Kaaraayvaz scRNA TNBC (epithelial, stroma, myoepithelial, monocyte, lymphocyte, TAM) and Nguyen scRNA normal breast (basal, myoepithelial, luminal 1-1, luminal 1-2 and luminal 2. **b,** METABRIC: METABRIC sample ids, clinical, TNBC subtype correlation, immune ESTIMATE and Xcell. **c,** CPTAC: CPTAC retrospective identifiers, clinical and TNBC subtype. **d,** MET500: MET500 identifiers, biopsy site, TNBC subtype correlation, immune ESTIMATE and Xcell.

Supplementary Data 3 (Microsoft Excel format): Single sample GSVA pathway analysis. Gene set enrichment for **a,** KEGG_BL1 b, KEGG_BL2 c, KEGG_LAR d, KEGG_M e, HALLMARK_BL1 f, HALLMARK_BL2 h, HALLARK_LAR i, HALLARK_M j, REACTOME_BL1 k, REACTOME_BL2 l, REACTOME_LAR m and REACTOME_M. GSVA is based on a non-parametric, unsupervised method for estimating variation of gene set enrichment. GS=Gene set name, SIZE=Number of genes in the gene set after filtering out those genes not in the expression dataset; ES=Enrichment score for the gene set; that is, the degree to which this gene set is overrepresented at the top or bottom of the ranked list of genes in the expression datase.; NES=Normalized enrichment score; that is, the enrichment score for the gene set after it has been normalized across analyzed gene sets; NOM p-value=Nominal p value; that is, the statistical significance of the enrichment score. The nominal p value is not adjusted for gene set size or multiple hypothesis testing; therefore, it is of limited use in comparing gene sets and FDR q-value=False discovery rate; that is, the estimated probability that the normalized enrichment score represents a false positive finding.

Supplementary Data 4 (Microsoft Excel format): TNBC subtype GISTIC copy number analysis.
a, BL1_Amp: GISTIC amplified copy number regions in BL1 subtype. **b,** BL1_Del: GISTIC deleted copy number regions in BL1 subtype. **c,** BL2_Amp: GISTIC amplified copy number regions in BL2 subtype. **d,** BL2_Del: GISTIC deleted copy number regions in BL2 subtype. **e,** LAR_Amp: GISTIC amplified copy number regions in LAR subtype. **f,** LAR_Del: GISTIC deleted copy number regions in LAR subtype. **g,** M_Amp: GISTIC amplified copy number regions in M subtype. **h,** M_Del: GISTIC deleted copy number regions in M subtype. **i,** TNBC_Amp: GISTIC amplified copy number regions in TNBC subtype. **j,** TNBC_Del:GISTIC deleted copy number regions in TNBC subtype.

Supplementary Data 5 (Microsoft Excel format): TNBC subtype-specific significant gene expression, methylation, RPPA, CPTAC protein and phosphoprotein results.

a, GE_BL1: Differentially expressed genes in BL1 subtype (LFC, average expression, p-value and FDR q-value. **b,** GE_BL2: Differentially expressed genes in BL2 subtype (LFC, average expression, p-value and FDR q-value. **c,** GE_M: Differentially expressed genes in M subtype (LFC, average expression, p-value and FDR q-value. **d,** GE_LAR: Differentially expressed genes in LAR subtype (LFC, average expression, p-value and FDR q-value. **e,** ME_BL1: Differentially expressed methylation probes in BL1 subtype (LFC, average expression, p-value and FDR q-value. **f,** ME_BL2: Differentially expressed methylation probes in BL2 subtype (LFC, average expression, p-value and FDR q-value. **g,** ME_M: Differentially expressed methylation probes in M subtype (LFC, average expression, p-value and FDR q-value. **h,** ME_LAR: Differentially expressed methylation probes in LAR subtype (LFC, average expression, p-value and FDR q-value. **i,** RPPA_BL1: Differentially expressed protein in BL1 subtype (LFC, average expression, p-value and FDR q-value. **j,** RPPA_BL2: Differentially expressed protein in BL2 subtype (LFC, average expression, p-value and FDR q-value. **k,** RPPA_M: Differentially expressed protein in M subtype (LFC, average expression, p-value and FDR q-value. **l,** RPPA_LAR: Differentially expressed protein in LAR subtype (LFC, average expression, p-value and FDR q-value. **m,** CPTAC_BL1: Differentially expressed protein in BL1 subtype (LFC, average expression, p-value and FDR q-value. **n,** CPTAC_phospho_BL1: Differentially expressed phospho-protein in BL1 subtype **o,** CPTAC_BL2: Differentially expressed protein in BL2 subtype (LFC, average expression, p-value and FDR q-value. **p,** CPTAC_phospho_BL2: CPTAC_phospho_M: Differentially expressed phospho-protein in BL2 subtype **q,** CPTAC_LAR: Differentially expressed protein in LAR subtype (LFC, average expression, p-value and FDR q-value. **r,** CPTAC_phospho_LAR: CPTAC_phospho_M: Differentially expressed phospho-protein in LAR subtype. **s,** CPTAC_M: Differentially expressed protein in M subtype (LFC, average expression, p-value and FDR q-value. **t,** CPTAC_phospho_M: Differentially expressed phospho-protein in M subtype. All statistical results determined by modified T-test from limma package corrected for multiple hypotheses.

Supplementary Data 6 (Microsoft Excel format): Cell line subtyping and genetic and pharmacological dependencies from DepMap GDSC and PDTX models. a, DepMap filter: Identification of TNBC cell models from DepMap. **b,** DepMap TNBC subtype: Subtyping correlations for DepMap cell lines. **c,** DepMap RNAi result: Subtype specific dependencies (negative T values) and significance in DepMap RNAi whole genome screen. d**,** DepMapsgRNA result: Subtype specific dependencies (negative T values) and significance in DepMap cRISPR whole genome screen. **e,** GDSC_TNBCtype: Subtyping correlations for GDSC cell lines. **f,** GDSC_result: Subtype specific dependencies (negative T values) and significance in GDSC pharmacological screen. **g,** PDTX_TNBC: Identification of TNBC PDTX cell models. **h,** PDTX_TNBCtype: Subtyping correlations for PDTX cell lines. **i,** PDTX_result: Subtype specific dependencies (negative T-values) and significance in PDTX pharmacological screen.

Supplementary Data 7 (Microsoft Excel format): DNA methylation ELMER analysis. a, TCGA samples and subtypes. TCGA barcode identifiers and respective TNBC subtypes. b, ELMER analysis results. The column "ELMER Analysis" shows in which analysis the pairs probe-gene were identified. For each differently methylated probe, ELMER predicts anti-correlated target genes. The columns "Raw-p" and "FDR" shows the results for the gene expression t-test analysis between the groups shown in "ELMER Analysis" column. **c,** ATAC-seq peaks – genes ATAC-seq peaks overlapping CpGs from table A and linked predicted target genes.
