# Supplementary material for: Multi-omics analysis identifies therapeutic vulnerabilities in triple-negative breast cancer subtypes
Source: Nat Commun. 2021 Nov 1;12:6276. doi: 10.1038/s41467-021-26502-6 (PMC8560912; doi:10.1038/s41467-021-26502-6)
Supplement: Supplementary file 11 — Reporting Summary [file 41467_2021_26502_MOESM11_ESM.pdf]

nature research

Corresponding author(s): X. Steven Chen and Brian D. Lehmann

Last updated by author(s): 2021/09/09

Reporting Summary

Nature Research wishes to improve the reproducibility of the work that we publish. This form provides structure for consistency and transparency in reporting. For further information on Nature Research policies, see our Editorial Policies and the Editorial Policy Checklist.

Statistics

For all statistical analyses, confirm that the following items are present in the figure legend, table legend, main text, or Methods section.

n/a	Confirmed	
☐	☒	The exact sample size (*n*) for each experimental group/condition, given as a discrete number and unit of measurement
☐	☒	A statement on whether measurements were taken from distinct samples or whether the same sample was measured repeatedly
☐	☒	The statistical test(s) used AND whether they are one- or two-sided<br>*Only common tests should be described solely by name; describe more complex techniques in the Methods section.*
☐	☒	A description of all covariates tested
☐	☒	A description of any assumptions or corrections, such as tests of normality and adjustment for multiple comparisons
☐	☒	A full description of the statistical parameters including central tendency (e.g. means) or other basic estimates (e.g. regression coefficient) AND variation (e.g. standard deviation) or associated estimates of uncertainty (e.g. confidence intervals)
☐	☒	For null hypothesis testing, the test statistic (e.g. *F*, *t*, *r*) with confidence intervals, effect sizes, degrees of freedom and *P* value noted<br>*Give P values as exact values whenever suitable.*
☒	☐	For Bayesian analysis, information on the choice of priors and Markov chain Monte Carlo settings
☒	☐	For hierarchical and complex designs, identification of the appropriate level for tests and full reporting of outcomes
☒	☐	Estimates of effect sizes (e.g. Cohen's *d*, Pearson's *r*), indicating how they were calculated

Our web collection on statistics for biologists contains articles on many of the points above.

Software and code

Policy information about availability of computer code

Data collection	TCGA data: NIH Genomic Data Commons (GDC), https://gdc.cancer.gov/. Accessed using TCGAbiolinks R package (v2.11.1) (Colaprico et al. 2016) [PMID:26704973], http://bioconductor.org/packages/TCGAbiolinks/ in May 2018.<br>CPTAC data: LinkedOmics [PMID: 29136207], http://linkedomics.org/data_download/TCGA-BRCA/. Manually accessed.<br>Public MC3 MAF: [PMID:29596782], https://gdc.cancer.gov/about-data/publications/mc3-2017. Manually accessed.<br>MET500: Metastatic solid tumors [PMID:28783718], Database of Genotypes and Phenotypes (dbGaP) accession number phs000673.v2.p1. Manually accessed.<br>METABRIC: [PMID: 22522925], EGAS00000000083. Manually accessed.<br>BRCA Single Cell RNA expression: [PMID: 29795293], GEO: GSE113197. Manually accessed.<br>BRCA Single Cell RNA expression: [PMID: 30181541], GEO: GSE118390. Manually accessed.<br>DepMap: [PMID:28753430], https://depmap.org/portal/download/. Manually accessed.<br>Breast Cancer PDTX Encyclopaedia: [PMID:27641504], https://caldaslab.cruk.cam.ac.uk/bcape/. Manually accessed.<br>Genomics of Drug Sensitivity in Cancer (GDSC): [PMID:23180760], https://www.cancerrxgene.org. Manually accessed.<br>Cancer Cell Line Encyclopedia (CCLE): [PMID: 31068700], https://portals.broadinstitute.org/ccle. Manually accessed.
Data analysis	All analysis: R 3.5.0, (R Foundation for Statistical Computing 2020), https://www.R-project.org<br>All analysis plots: ggplot2 R package (v.2.2.1), (Wickham 2016), https://CRAN.R-project.org/package=ggplot2<br>Heatmap plots: ComplexHeatmap R package (v.2.8.0), (Gu et al. 2016) [PMID:27207943], https://bioconductor.org/packages/ComplexHeatmap/<br>TCGA data downloading: R/Bioconductor package TCGAbiolinks (v.2.9.5), https://bioconductor.org/packages/release/bioc/html/TCGAbiolinks.html<br>A Subtyping for Triple-Negative Breast Cancer: TNBCtype, (Chen et al. 2012) [PMID:22872785], http://cbc.mc.vanderbilt.edu/tnbc/<br>Consensus clustering: R packages ConsensusClusterPlus (v.1.54.0) and CancerSubtypes (v.1.16.0)<br>Non-negative matrix factorization: Maftools R package(v.2.8.0), (Mayakonda et al. 2018) [PMID:30341162] https://bioconductor.org/

packages/release/bioc/html/maftools.html
Deconvolution of immune cell type composition: xCell (v.1.1.0), (Aran et al. 2017) [PMID: 29141660], https://github.com/dviraran/xCell.
Enhancer linking by methylation/expression relationships (ELMER) analysis: ELMER (v.2.14.0), (Silva et al. 2019) [PMID:30364927] http://bioconductor.org/packages/ELMER/.
Copy number variant calling: GISTIC2.0 (v.2.0.23), (Mermel et al. 2011) [PMID:21527027], ftp://ftp.broadinstitute.org/pub/GISTIC2.0/GISTIC_2_0_23.tar.gz
Estimation of tumor purity, stromal and immune scores : ESTIMATE (v.1.0.13), (Yoshihara et al. 2013) [PMID:24113773], https://bioinformatics.mdanderson.org/public-software/estimate/
Gene Set Variation. Analysis: GSVA R package (v1.40.1), (Hänzelmann et al. 2013) [PMID: 23323831], https://bioconductor.org/packages/release/bioc/html/GSVA.html
TNBC subtype association testing for omics data: R packages sva (v.3.40.0) and limma (v.3.48.3)
RNA-seq analysis: STAR (v.2.5.0) [PMID:23104886], featureCount (v.1.5.0) [PMID:24227677]
Single-cell RNAseq analysis: R package Seruat (v.3.2.3), MuSiC (v.0.2.0) https://github.com/xuranw/MuSiC
ChIP-seq analysis: bowtie (v.1.2.2) [PMID:MACS (v.2.1.1)[PMID:24743991], deepTools (v.3.5.01) [PMID:20110278],bedtools (v.2.30.0), ChIPseeker (v.1.28.3) [PMID:25765347] and Integrative Genomics Viewer (2.4.16)
Immunohistochemistry analysis: Qupath (v.0.3.0) PMID[29203879]
Flow cytometric analysis: flowCore (v.2.2.0), flowStats (v.4.2.0), R packages ggcyto (v.1.18.0), ggridges (v.0.5.3)
Custom R scripts: available upon request.

For manuscripts utilizing custom algorithms or software that are central to the research but not yet described in published literature, software must be made available to editors and reviewers. We strongly encourage code deposition in a community repository (e.g. GitHub). See the Nature Research guidelines for submitting code & software for further information.

Data

Policy information about availability of data

All manuscripts must include a data availability statement. This statement should provide the following information, where applicable:

- Accession codes, unique identifiers, or web links for publicly available datasets
- A list of figures that have associated raw data
- A description of any restrictions on data availability

TCGA -omics data is available on the NIH Genomic Data Commons (GDC).
CPTAC normalized datasets (CPTAC RNA, HS_CPTAC_BRCA_2018_RNA_GENE.cct.txt;CPTAC proteome, HS_CPTAC_BRCA_2018_Phosphoproteome_Ratio_Norm_Site.cct.txt;CPTAC phosphoproteome, HS_CPTAC_BRCA_2018_Phosphoproteome_Ratio_Norm_Gene_median.cct.txt; CPTAC Clinical metadata, HS_CPTAC_BRCA_2018_CLI.tsi.txt) are publicly available through the LinkedOmics publicly available data portal (http://linkedomics.org/data_download/TCGA-BRCA/).
METABRIC associated genotype and expression data is available at the European Genome-Phenome Archive (http://www.ebi.ac.uk/ega/), which is hosted by the European Bioinformatics Institute, under accession number EGAS00000000083 (https://ega-archive.org/studies/EGAS00000000083).
MET500 Sequencing data from the 500 patients is available from the dbGaP under accession number phs000673.v2.p1 (https://www.ncbi.nlm.nih.gov/projects/gap/cgi-bin/study.cgi?study_id=phs000673.v2.p1). The MET500 web portal is available at http://met500.path.med.umich.edu.
TCGA mutation data is available at MC3 Public MAF - mc3.v0.2.8.PUBLIC.maf.gz. Raw sequencing files are available at Sequence Read Archive (SRA) PRJNA647007 (https://www.ncbi.nlm.nih.gov/bioproject/PRJNA647007).
Single-cell RNA-seq data is available in the Gene Expression Omnibus under accession code GSE113197 (https://www.ncbi.nlm.nih.gov/geo/query/acc.cgi?acc=GSE113197) and GSE118390 (https://www.ncbi.nlm.nih.gov/geo/query/acc.cgi?acc=GSE118390).
CCLE cell lines gene expression data was obtained from: https://portals.broadinstitute.org/ccle. Cell line annotations (DepMap-2018q4-celllines.csv) were obtained from DepMap (https://depmap.org/portal/download/).
DepMap RNAi (D2_combined_gene_dep_scores.csv) and CRISPR (CRISPR_gene_effect.csv) cell lines dependencies are available from the Broad DepMap https://depmap.org/portal/download/.
Gene expression data for cell lines (GDS_v17_TNBC_IC50.csv) from the Genomics of Drug Sensitivity in Cancer (GDSC) is available in https://www.cancerrxgene.org.
Gene expression data for cell lines from patient-derived tumor xenograft (PDTX) is available in Breast Cancer PDTX Encyclopaedia https://caldaslab.cruk.cam.ac.uk/bcape/.
Full immunoblot scans available in source data.
All other data supporting the findings of this study are available within the article, its supplementary information files and the source data provided with this paper.
Raw sequencing files of TNBC cell lines are available at https://www.ncbi.nlm.nih.gov/bioproject/PRJNA647007

Field-specific reporting

Please select the one below that is the best fit for your research. If you are not sure, read the appropriate sections before making your selection.

☒ Life sciences ☐ Behavioural & social sciences ☐ Ecological, evolutionary & environmental sciences

For a reference copy of the document with all sections, see nature.com/documents/nr-reporting-summary-flat.pdf

Life sciences study design

All studies must disclose on these points even when the disclosure is negative.

Sample size

The exploratory analysis was performed on all available TNBC tumors from TCGA and CPTAC.

We considered as sufficient a minimum sample size of 3 replicates for cell lines treated with inhibitors for our RNA-Seq analysis. This number is satisfactory to achieve a power between 0.8 and 1 (one e.g., 100% detection of differentially expressed genes) as mentioned in Ching, et. al, Power analysis and sample size estimation for RNA-Seq differential expression, RNA, 2014

Data exclusions	No exclusion criteria were pre-established outside of clinical-genomic filtering to identify TNBC tumors from other breast cancers in TCGA, CPTAC, METABRIC and MET500 datasets.
Replication	Immune cell estimation and gene expression differences in TCGA were validated in METABRIC and MET500 datasets and shown in Extended Data Fig.2. Experiments were independently repeated a minimum of two times and reliably reproduced across multiple cell lines indicated in the figure legends.<br>ChIP seq (two replicates)<br>Flow cytometry (three replicates)<br>Immunoblots (representative of two replicates)<br>Viability experiments (3 independent replicates)<br>IHC staining (3 independent cores)<br>Mice (10 independent tumors)
Randomization	Syngeneic mice bearing 4T1 xenografts were randomized into control and treatment groups when tumors reached approximately 50mm3 so that the median and variance of tumors were similar among treatment groups. T<br>he order in which cell lines were seeded and treated for in-vitro experiments was done at random.<br>For cell line xenograft injections, the order in which injections were performed was random.
Blinding	Blinded evaluation of tumor immune microenvironment (TIME) classification was performed by two expert pathologists (M.E.S and P.I.G-E) independently on random H&E images from TCGA. Automated semiquantitative scoring of CD3+ and HLA-A positive cells were determined on digitized images by a blinded pathologist using QuPath software. Blinding was not done for the mouse experiments as each group received different treatments.<br>For in vivo tumor xenograft experiments, injections and tumor volume measurements were performed with experimenter blinded for treatment. For the in vitro experiments not listed here, investigators were not blinded because the results of measurements or sequencing experiments were not affected by knowledge of cell line identities.

Reporting for specific materials, systems and methods

We require information from authors about some types of materials, experimental systems and methods used in many studies. Here, indicate whether each material, system or method listed is relevant to your study. If you are not sure if a list item applies to your research, read the appropriate section before selecting a response.

Materials & experimental systems

n/a	Involved in the study
☐	☒ Antibodies
☐	☒ Eukaryotic cell lines
☒	☐ Palaeontology and archaeology
☐	☒ Animals and other organisms
☒	☐ Human research participants
☒	☐ Clinical data
☒	☐ Dual use research of concern

Methods

n/a	Involved in the study
☒	☐ ChIP-seq
☐	☒ Flow cytometry
☒	☐ MRI-based neuroimaging

Antibodies

Antibodies used	anti-H2-Kd (SF1-1.1, 1:1000, Biolegend)<br>anti-Ki67 (1:250, Catalog #12202S, Cell Signaling Technology, Danvers, MA)<br>anti-caspase3 (1:300, Catalog # 9664S, Cell Signaling Technology, Danvers, MA)<br>Tri-Methyl-Histone H3K27 (1:300, Catalog 9733, Cell Signaling Technology, Danvers, MA).<br>HLA-A antibody (Santa Cruz Biotechnology, sc-365485; clone C6,dilution 1:1300)<br>anti-histone 3 (1:5000, Abcam, ab1791)<br>HLA-A/B/C (1:1000, Santa Cruz, sc-52810)<br>anti-GAPDH (1:5000, EMD Millapore, MAB374)<br>anti-vinculin (1:2000, ThermoFisher, 700062).<br>Goat anti-Mouse IgG, HRP-conjugated secondary, Thermo Fischer Scientific, Cat# 31432; RRID:AB_228302;<br>Goat anti-Rabbit IgG, HRP-conjugated secondary, Thermo Fischer Scientific, Cat# 31462, RRID:AB_228338
Validation	All antibodies were tested by the manufacturer for relevant applications to ensure specific staining to the antigen without cross-reactivity. All antibodies for immunohistochemistry were optimized on FFPE sections using relevant target species from tissues known to express target antigen.<br>Ki-67 (D3B5, Cell Signaling Technology) Rabbit mAb (Mouse Preferred; IHC Formulated) recognizes endogenous levels of murine Ki-67 protein. IHC staining has been validated on proliferating cells in mouse spleen and colon and negative staining in non-proliferative mouse heart tissue. We also observed positivity outside of necrotic tumor core (Supplementary Figure 12).<br><br>Tri-Methyl-Histone H3K27 (Catalog #9733, Cell Signaling Technology) antibody detects endogenous levels of histone H3 only when tri-methylated on Lys27. The antibody does not cross-react with non-methylated, mono-methylated or di-methylated Lys27. In

addition, the antibody does not cross-react with mono-methylated, di-methylated or tri-methylated histone H3 at Lys4, Lys9, Lys36 or Histone H4 at Lys20. Monoclonal antibody is produced by immunizing animals with a synthetic peptide corresponding to the amino terminus of histone H3 in which Lys27 is tri-methylated. The trimethyl lysine mark is added to histone 3 by the enzymatic activity of EZH2 in complex with polycomb repressor 2 components EED and SUZ12. We validated this antibody by observing the relative decreases in signal intensity following treatment with two EZH2 inhibitors (tazemetostat and CP1205) or an EED inhibitor (Figure 5).

HLA-A antibody (Santa Cruz Biotechnology, sc-365485; clone C6) is a mouse monoclonal antibody raised to an epitope mapping between amino acids 61-93 within an internal region of HLA-A of human origin and recommended for detection of a broad range of HLA antigens of human origin by WB, IP, IF, IHC(P) and ELISA. The antibody has been validated by immunoblot in U-87 MG, THP-1 and NCI-H292 whole cell lystates and by IHC of human spleen tissue showing membrane staining of cells in white pulp and cells in red pulp.
The anti-H2-Kd (SF1-1.1, 1:1000, Biolegend) antibody recognizes the mouse d haplotype and is weakly cross-reactive with H-2k but does not cross-react with other haplotypes (b, j, p, q, s, v). Clone SF1-1.1 recognizes the a3 domain of Kd. The antibody has been extensivity cited and validated by flow cytometry of Balb/c mouse splenocytes.

Anti-caspase3 (Catalog # 9664S, Cell Signaling Technology) is a monoclonal antibody is produced by immunizing animals with a synthetic peptide corresponding to amino-terminal residues adjacent to Asp175 of human caspase-3. The antibody detects endogenous levels of the large fragment (17/19 kDa) of activated caspase-3 resulting from cleavage adjacent to Asp175. This has been validated by immunoblot in NIH/3T3 (mouse), and Jurkat (human) cells, untreated or treated with staurosporine (1uM, 3hrs) or etoposide. Additional validation has been done by immunohistochemical analysis of paraffin-embedded mouse embryo in the presence of control peptide or Cleaved Caspase-3 (Asp175) Blocking Peptide.
Anti-histone 3 (Abcam, ab1791) antibody is a ChIP grade anti H3 antibody that reacts with both human and mouse multiple variants of H3 such as H3.1, H3.2 and H3.3. The 17kDa reactivity with H3 has been extensively validated in A431 (Human epithelial carcinoma cell line),Jurkat (Human T cell lymphoblast-like cell line) Whole Cell Lysate and HEK293 (Human embryonic kidney cell line) whole cell lysates treated with recombinant Human Histone H3 peptide.
HLA-A/B/C (Santa Cruz, LY5.1,sc-52810) is a mouse monoclonal antibody raised against purified HLA-A/B/C of human origin. a. This antibody is recommended for Western Blotting, immunoprecipitation and immunofluorescence. The antibody has been validated in HeLa 293T or human PBL whole cell lysates.
Anti-Glyceraldehyde-3-Phosphate Dehydrogenase (GAPDH) Antibody, clone 6C5 is a well published and extensively characterized monoclonal antibody. This purified mAb detects Glyceraldehyde-3-Phosphate Dehydrogenase (GAPDH) & has been published & validated for use in ELISA, IP, IC, IF, IH & WB. It recognizes a 36kDa band of the reduced monomer. Non-reduced GAPDH runs as a 146kDa tetramer. Specificity: Glyceraldehyde-3-phosphate dehydrogenase (GAPDH) from skeletal muscle. Antibody also recognizes cardiac GAPDH. GAPDH enzyme is detected in many non-muscle cells lines including HeLa, HCT-116 cells, U937 and THP-1 cells among others. For quality assurance this antibody was evaluated by Western Blot on A431 lysates.

Anti-Vinculin. This antibody reacts with Human Vinculin. Based on sequence similarity, reactivity to chimpanzee, Rhesus monkey, swine, equine, mouse, rat, bovine, and chicken is expected. Immunogen sequence:
IRGALAEARKIAELCDDPKERDDILRSLGEISALTSKLADLRRQGKGDSPEARALAKQVATALQNLQTKTNRAVANSRPAKAAVHLEGKIEQAQRWIDNPTV DDRGVGQAAIRGLVAEGHRLANVMMGPYRQDLLAKCDRVDQLTAQLADLAARGEGESPQARALASQLQDSLKDLKARMQEAM. Test material for western blotting/immunofluorescence: HeLa cells. This Antibody was verified by Relative expression to ensure that the antibody binds to the antigen stated.

Goat anti-Mouse IgG, HRP-conjugated secondary - Antibody Specificity: This antibody reacts with whole molecule of mouse IgG and with the light chains of other mouse immunoglobulins. No antibody was detected against non-immunoglobulin serum proteins. The antibody has been tested by ELISA and/or solid-phase adsorbed to ensure minimal cross-reaction with human, bovine, and horse serum proteins. However, this antibody may cross-react with immunoglobulins from other species.

Goat anti-Rabbit IgG, HRP-conjugated secondary - Antibody Specificity: This antibody reacts with the heavy chains of rabbit IgG and with the light chains common to most rabbit immunoglobulins. No antibody was detected against non-immunoglobulin serum proteins. The product has been tested by ELISA and/or solid-phase adsorbed to ensure minimal cross-reaction with human serum proteins. However, this antibody may cross-react with immunoglobulins from other species.

Eukaryotic cell lines

Policy information about cell lines

Cell line source(s)	TNBC cell lines were purchased from ATCC (BT549, HS578T. MDAMB468, MDAMB436, HCC1143, HCC1806 and HCC1937) and DSMZ (CAL-51, CAL120 and HDQP1) in 2010. 4T1 cells were kindly provided by Dr. Justin Balko (pur
Authentication	Cell lines were authenticated by short-tandem repeat profiling (March 2011).
Mycoplasma contamination	Cell lines were regularly tested and verified to be mycoplasma-negative by PCR analysis (Lonza).
Commonly misidentified lines (See ICLAC register)	No commonly misidentified cell lines were used in this study.

Animals and other organisms

Policy information about studies involving animals; ARRIVE guidelines recommended for reporting animal research

Laboratory animals	Wild-type BALB/c female mice age 6-8 weeks.<br>Female BALB/c 6-8 weeks were obtained from The Jackson Laboratory (000651). Mice were housed with 12 h light-dark cycles at

	20-26C and 30-70% humidity, in ventilated cages with five mice per cage, and constant access to food and water.
Wild animals	The study did not involve wild animals.
Field-collected samples	The study did not involve samples collected from the field.
Ethics oversight	Mice were housed and treated in accordance with NIH guidelines and protocols approved by the Institutional Animal Care and Use Committee at the Vanderbilt University Medical Center. (IACUC; protocol M1900101-00)

Note that full information on the approval of the study protocol must also be provided in the manuscript.

Flow Cytometry

Plots

Confirm that:

☒ The axis labels state the marker and fluorochrome used (e.g. CD4-FITC).

☒ The axis scales are clearly visible. Include numbers along axes only for bottom left plot of group (a 'group' is an analysis of identical markers).

☒ All plots are contour plots with outliers or pseudocolor plots.

☒ A numerical value for number of cells or percentage (with statistics) is provided.

Methodology

Sample preparation	TNBC cell lines were treated with inhibitors for five days and cells were harvested with accutase, quenched with media and washed in phosphate buffered saline.
Instrument	Flow cytometry was performed on a Attune NxT Flow Cytometer (Thermo Fisher)
Software	Raw .fcs files were processed in R (flowCore and flowStats), corrected with compensation and plotted with R packages ggcyto and ggridges.
Cell population abundance	Median fluorescence intensity of treated cell populations were used to determine fold-change compared to median fluorescence intensity of untreated cells and averaged from three independent replicates.
Gating strategy	The following steps describe the gating strategy depicted in Figs. 5c and i and extended data Fig. 6:<br>1. FSC/SCC exclusion of particles with FCC<30k.<br>2. Negative staining for Zombie Violet to remove dead cells.<br>3. Positive staining for PE conjugated H2-Kd (mouse) or HLA-A/B/C (human).

☒ Tick this box to confirm that a figure exemplifying the gating strategy is provided in the Supplementary Information.
